# The Impact of Information Relevancy and Interactivity on Intensivists’ Trust in a Machine Learning–Based Bacteremia Prediction System: Simulation Study

**DOI:** 10.2196/56924

**Published:** 2024-08-01

**Authors:** Omer Katzburg, Michael Roimi, Amit Frenkel, Roy Ilan, Yuval Bitan

**Affiliations:** 1 Department of Health Policy and Management, Ben-Gurion University of the Negev, Be'er Sheva, Israel; 2 General Intensive Care Unit, Rambam Medical Center, Haifa, Israel; 3 General Intensive Care Unit, Soroka Medical Center, Be'er Sheva, Israel

**Keywords:** user-interface design, user-interface designs, user interface, human-automation interaction, human-automation interactions, trust in automation, automation, human-computer interaction, human-computer interactions, human-ML, human-ML interaction, human-ML interactions, decision making, decision support system, clinical decision support, decision support, decision support systems, machine learning, ML, artificial intelligence, AI, machine learning algorithm, machine learning algorithms, digitization, digitization of information

## Abstract

**Background:**

The exponential growth in computing power and the increasing digitization of information have substantially advanced the machine learning (ML) research field. However, ML algorithms are often considered “black boxes,” and this fosters distrust. In medical domains, in which mistakes can result in fatal outcomes, practitioners may be especially reluctant to trust ML algorithms.

**Objective:**

The aim of this study is to explore the effect of user-interface design features on intensivists’ trust in an ML-based clinical decision support system.

**Methods:**

A total of 47 physicians from critical care specialties were presented with 3 patient cases of bacteremia in the setting of an ML-based simulation system. Three conditions of the simulation were tested according to combinations of information relevancy and interactivity. Participants’ trust in the system was assessed by their agreement with the system’s prediction and a postexperiment questionnaire. Linear regression models were applied to measure the effects.

**Results:**

Participants’ agreement with the system’s prediction did not differ according to the experimental conditions. However, in the postexperiment questionnaire, higher information relevancy ratings and interactivity ratings were associated with higher perceived trust in the system (*P*<.001 for both). The explicit visual presentation of the features of the ML algorithm on the user interface resulted in lower trust among the participants (*P*=.05).

**Conclusions:**

Information relevancy and interactivity features should be considered in the design of the user interface of ML-based clinical decision support systems to enhance intensivists’ trust. This study sheds light on the connection between information relevancy, interactivity, and trust in human-ML interaction, specifically in the intensive care unit environment.

## Introduction

### Overview

In the intensive care unit (ICU), intensivists make an extremely high number of decisions. For example, McKenzie et al [1] found that approximately 100 decisions are made every morning round. According to Ward et al [2], despite the continual increase in the number of ICUs, the number of intensivists remains about the same, resulting in an extremely high workload. The high rate of decision-making together with the continuous overload prompts the need for decision support tools.

Although machine learning (ML) algorithms and systems serving the medical community are continually increasing, their adoption into routine health care practice is not guaranteed [3]. One reason is the complexity of the algorithms, which often leads to clinicians’ lack of trust in such systems [4]. A multidisciplinary approach may enhance trust, by considering the human factor, the technological aspect, and the interaction between them [5]. This study examined 2 human-automation interaction features that emphasize the importance of the human factor in the design of ML-based clinical decision support systems (CDSSs).

### Clinical Decision Support Systems

To date, many CDSSs are categorized as “expert systems”—systems that try to imitate the way an ideal physician would think. These systems generate conclusions based on sets of rules [6]. In contrast, ML algorithms approach problems in the opposite way—they generate rules from historical data [6,7]. ML algorithms are currently being developed in almost every field of medicine and, in many instances, are already providing equal or even greater accuracy than physicians (eg, [8-10]). However, though ML CDSSs can enhance the quality of care, the adoption of such systems in all medical fields, and specifically in critical care, remains low [11].

In contrast to expert systems, ML algorithms are complex, and understanding and explaining the reasoning underlying them is often impossible [12]. Thus, ML algorithms are frequently considered black box algorithms. This fosters physicians’ distrust and skepticism of ML systems [13] and has been suggested as a major cause of the low rates of adoption and acceptance of these systems within the medical community [14]. Wrong decisions made by intensivists can result in severe and even fatal outcomes. Thus, they may be reluctant to share their decision-making responsibilities with black box CDSSs that they do not understand [11].

### Interpretable ML

As ML algorithms are developed to serve humans, human interaction with them must be considered. One approach to move from a “black box” to a “clear box” [15] lies in the growing field of interpretable ML [16-19]. Miller [20] offered an approach that combines artificial intelligence, social science, and human-computer interaction (HCI). He referred to “human–agent interaction” as the intersection of these 3 domains, including it as part of the interpretable ML field. Impressive work has been performed on interpretable ML in the HCI community (eg, [21-24]). Unfortunately, the ML community and the HCI community do not always work together [25]. This results in poor usability of many interpretable ML algorithms [20], yet opens an opportunity for HCI and interaction design researchers to seek means of enhancing trust in ML CDSSs [26].

### Human-Automation Trust

Parasuraman and Riley [27] defined automation as a technology that executes “a function that was previously carried out by a human.” This wide definition covers all kinds of machines, computers, and applications of artificial intelligence. Human-automation trust is a well-studied subject (eg, [28-34]). In the context of human social interactions, trust can be defined as “the willingness to be vulnerable to the actions of another person” [35]. Research has shown that humans perceive computers as social actors and may interact with them as they would with each other [36-38]. The interaction between humans and automated systems, or, in the context of this study, intensivists and black box algorithms, has also been shown to be substantially influenced by trust [31].

Although human-automation trust is being researched by many disciplines, no dominant model or approach has been determined for its measure. However, a well-accepted conclusion is that trust is not a standalone construct, but rather multidimensional [32]. In this study, we used the definition of Lee and See [29] for human-automation trust “an attitude that an agent will help achieve an individual’s goals in a situation characterized by uncertainty and vulnerability.” This definition corresponds well with the interaction between intensivists and ML CDSSs, even though the ICU environment is characterized by high levels of both uncertainty and vulnerability.

**Figure 1.**
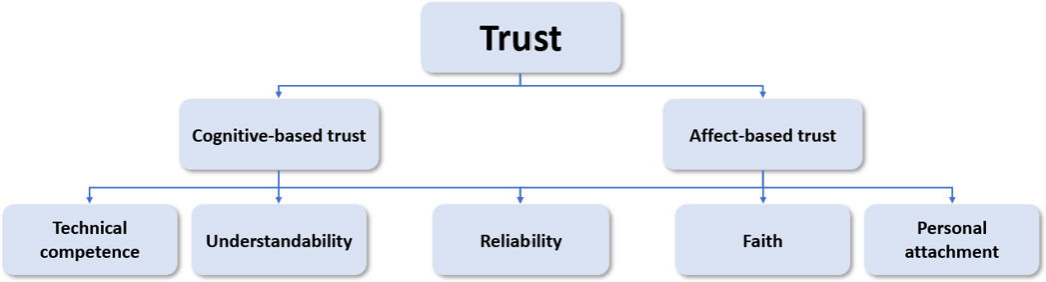
Madsen and Gregor’s [30] human-computer trust model.

According to Madsen and Gregor [30], human-computer trust is comprised of 2 main dimensions—cognition-based trust (CBT) and affect-based trust (ABT). CBT is based on the user’s intellectual perceptions of the system’s characteristics, while ABT is based on the user’s emotional responses to the system. The 2 dimensions can be further subdivided. CBT is comprised of the understandability of the system and the technical competence of the system, whereas ABT is comprised of faith, personal attachment, and reliability. Madsen and Gregor [30] note that reliability was also found to influence CBT, although its influence on ABT is stronger. The researchers suggested a questionnaire for measuring trust, which we implemented in this study (Figure 1).

### Aim

The primary aim of this study was to investigate the influence of elements of the user interface (UI) design on intensivists’ trust in ML-based CDSSs (“black-box”–based algorithms). From the many UI elements that can be modified, the 2 that were chosen and compared are information relevancy and interface interactivity.

The literature is abundant regarding information relevancy, interactivity, and trust, as well as the influence of the 2 former factors on the latter. However, to the best of our knowledge, no research has assessed connections between information relevancy, interactivity, and trust in the context of human-ML interaction, specifically in the context of the ICU environment.

### Hypothesis 1: Information Relevancy

Information relevancy concerns the degree to which users perceive that the information content of a system meets their needs [39]. This factor was found to positively influence user satisfaction with websites [39,40] and users’ trust in health infomediaries [41]. Relevant information has been found to be an attribute that is more crucial for users than usability and convenient use of the system [42]. Considering the above, our hypothesis is as follows:

- *Higher levels of information relevancy will lead to higher levels of trust in the system.* [H1]

### Hypothesis 2: Interface Interactivity

Interactivity can be defined in various ways. For this study, we used a common definition by Steuer [43]—“the extent to which users can participate in modifying the format and content of a mediated environment in real time.” Interactivity is considered to strongly influence users’ experiences during the interaction [44] and is key to the success of e-commerce websites [45-47]. Interactivity was found to increase users’ trust in websites in general and specifically in e-commerce, mobile commerce [48,49], and brand loyalty [44]. Although most of the literature on interactivity has focused on e-commerce trust and intentions to use websites, we expected greater interface interactivity to positively influence the interaction between ML CDSSs and intensivists, and to enhance their trust. Considering the above, our hypothesis is as follows:

- *Higher levels of interface interactivity will lead to higher levels of trust in the system.* [H2]

## Methods

### Overview

To test the hypotheses, a laboratory experiment with 3 conditions was designed. This enabled testing the effects of information relevancy and interactivity on intensivists’ trust in a simulated ML-based bacteremia prediction system. Bacteremia is a common phenomenon in ICUs, that clinicians need to identify and respond to [50]. Thus, a decision support system that assists clinicians in identifying this condition can serve as a good reference for generalizing and deriving implications for the UI design of many ML-based CDSSs. Each experimental condition was characterized by a different set of UI. The effects were measured with both a behavioral measure (the participants’ decisions that were captured by the simulation software) and a postexperiment questionnaire that captured their perceived understanding of the system.

### Participants

The participants were 47 physicians (female: n=14; male: n=33) from critical care specialties of 5 tertiary hospitals in Israel. They were recruited through a convenience sample of on-duty physicians and were free to withdraw from the study at any time. The experiment was conducted for 1 month, between the first and second COVID-19 lockdowns in Israel. All the participants were compensated with a gift card (US $15) and there were no exclusion criteria except for being a critical care physician.

### Ethical Considerations

This research complied with the American Psychological Association Code of Ethics and was approved by the institutional review board at Ben-Gurion University of the Negev (21-12-19). Informed consent was obtained from each participant.

### Experimental Design

To test the hypotheses, a 2×2 (relevant/nonrelevant×interactive/noninteractive) between-subjects fractional factorial experiment was designed. The experiment included 3 conditions (as shown in Table 1). The 15‐16 participants were randomly assigned to 1 of the 3 conditions; the duration of their performance was not limited. A total of 3 clinical cases of patients who were hospitalized in an ICU with medical conditions implying bacteremia onset were extracted. The presentations of these cases were designed by 3 experienced intensivists to provide accurate context.

**Table 1.** The experimental conditions.

	Noninteractive	Interactive
Nonrelevant information	1	—^a^
Relevant information	2	3

a Not tested.

### Apparatus and Stimuli

**Figure 2.**
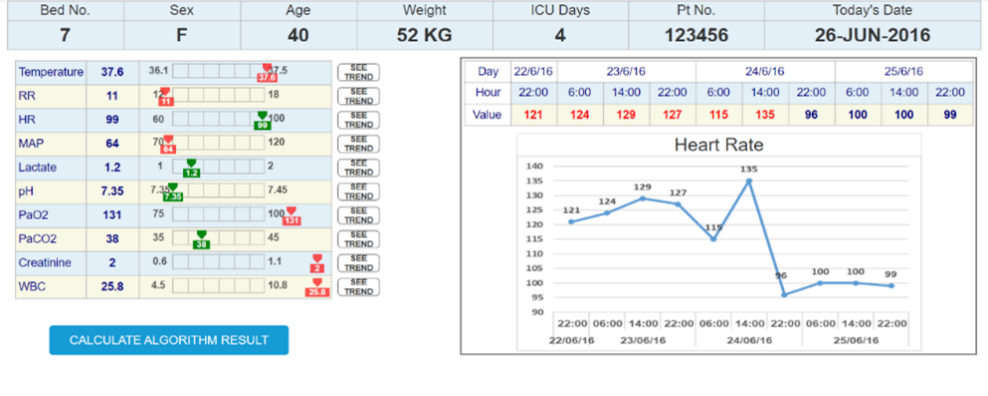
The right section shows the time-series chart, and the left section shows the patient's current clinical measures. HR: heart rate; ICU: intensive care unit; MAP: mean arterial pressure; RR: respiratory rate; WBC: white blood cell count.

**Figure 3.**
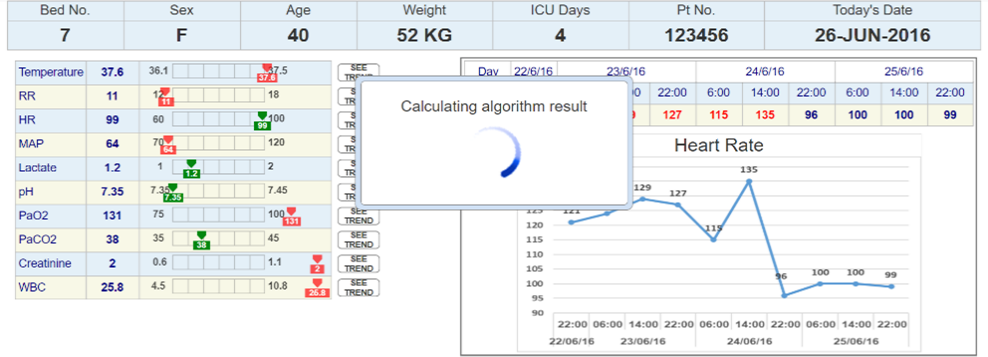
The bacteremia prediction system calculates the result. HR: heart rate; ICU: intensive care unit; MAP: mean arterial pressure; RR: respiratory rate; WBC: white blood cell count.

**Figure 4.**
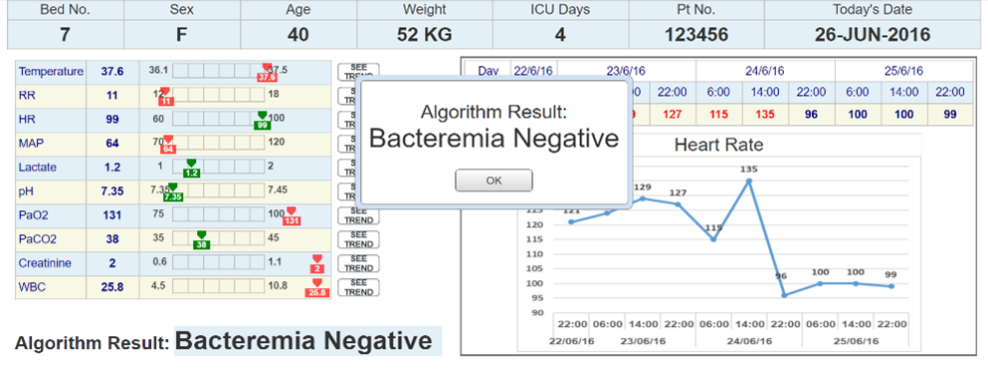
The bacteremia prediction system presents its prediction. HR: heart rate; ICU: intensive care unit; MAP: mean arterial pressure; RR: respiratory rate; WBC: white blood cell count.

A total of 3 UIs that represent 3 medical conditions were designed using Axure RP software (version 9.1; Axure Software Solutions, Inc). The interfaces were imitating an ML bacteremia prediction system. The system, which at the time of the study was still in its development stage, provides prediction and a list of the main features that were significant for the prediction algorithm. The right section of all the interfaces presented similar time-series charts. The charts included trends over time for the 10 clinical measures that are most related to bacteremia prediction. The information that was presented in the left section was manipulated to match the 3 conditions. An example of an interface (condition 2) is shown in Figures 2-4.

**Figure 5.**
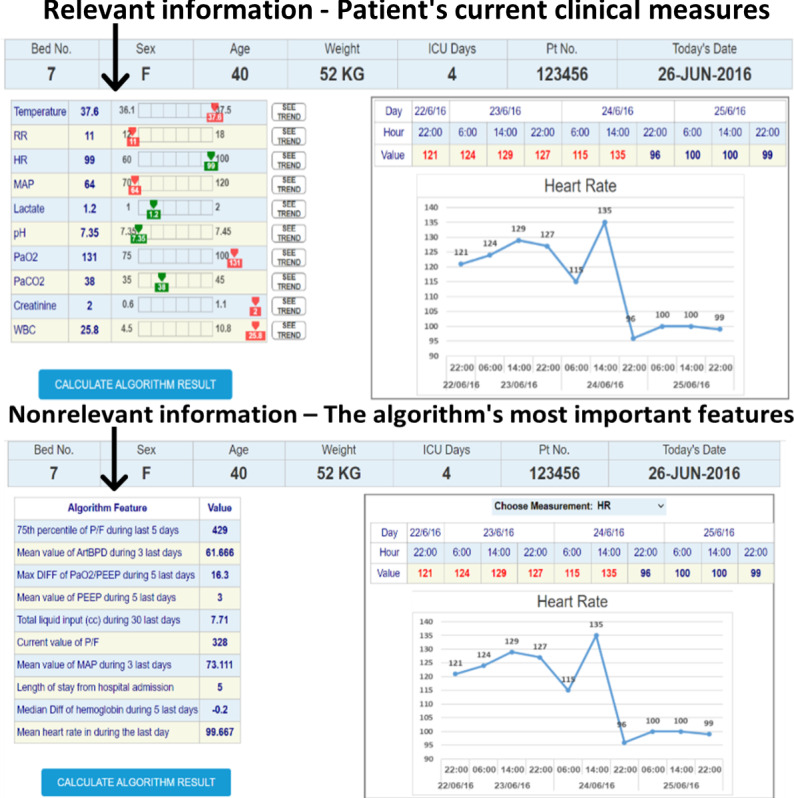
The relevant and nonrelevant conditions. The top frame shows the relevant information condition with the patient's current clinical measures; the bottom frame shows the nonrelevant information condition with the values of the algorithm's most important features. ArtBPD: arterial line blood pressure; HR: heart rate; ICU: intensive care unit; MAP: mean arterial pressure; PEEP: positive end-expiratory pressure: P/F ratio: PaO_2_/FIO_2_: oxygen arterial pressure to percentage of inspired oxygen ratio; RR: respiratory rate; WBC: white blood cell count.

The information relevancy level was set by the type of clinical measurements that were presented in a table in the left section of the chart. For the relevant information conditions, the information presented in the table comprised the current values of the same clinical measures that clinicians usually use to assess a patient’s condition. In addition, the normal range of each measure was presented. In the nonrelevant information condition, the information presented in the table comprised the values of the 10 features that were ranked as most important by the bacteremia ML prediction algorithm for making the prediction. Although these features were most significant for the prediction algorithm, they were not usually used by clinicians and, therefore, were considered nonrelevant (see Figure 5).

**Figure 6.**
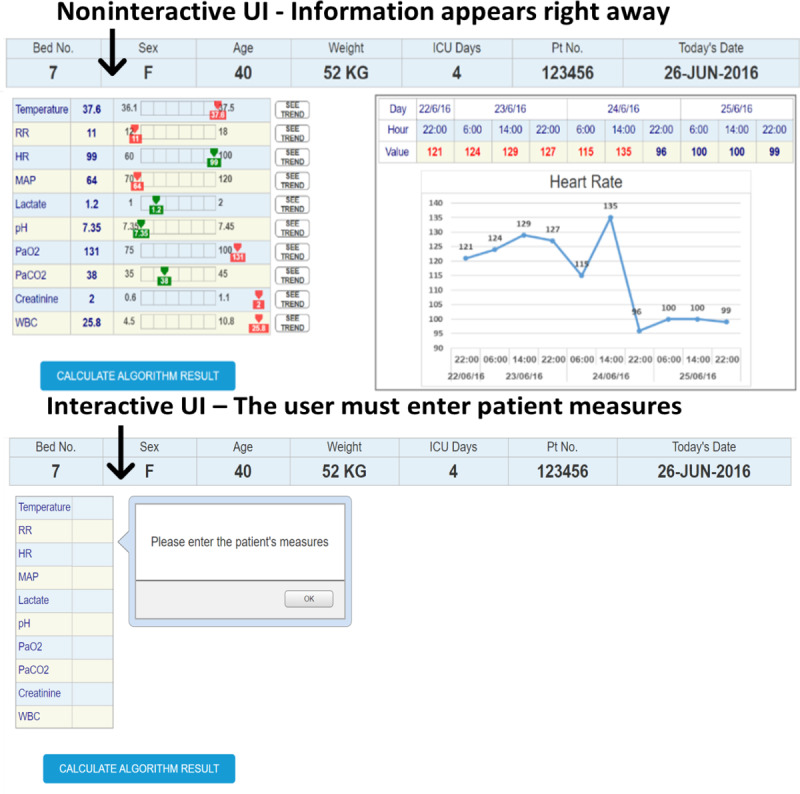
The interactive and noninteractive conditions. The top frame shows the noninteractive condition. The bottom frame shows the interactive condition, in which participants had to actively engage with the UI and provide the patient's current clinical measures, before they could explore the other charts and information. HR: heart rate; ICU: intensive care unit; MAP: mean arterial pressure; RR: respiratory rate; UI: user interface: WBC: white blood cell count.

The interface interaction level was set by the type of interaction that the participants were assigned with the UI. In the interactive condition, the participants were required to enter values of the patient’s current clinical measures (the values provided in the written clinical case) before they could explore the other charts and information. Entering and copying values to and from the patient record is a common task clinicians apply in a subset of the IT systems in the ICU. In the noninteractive conditions, the information about the patients appeared right away, and the participants could only explore the information and ask the system for its prediction (see Figure 6).

The fourth combination, nonrelevant information and interactivity, was not tested, as in the nonrelevant information condition, the information that was presented was of the features of the algorithm. Thus, including the algorithm features in the clinical case and entering them into the UI would seem unrealistic.

### Procedure

The participants were introduced to the purpose of the study and received an explanation about the ML bacteremia prediction system. They were then introduced to the simulation software, with the UI fitting the condition they were assigned. The participants were asked to first read the clinical case, and only then to explore the UI. After exploring the UI, they could click on the “calculate algorithm result” button to receive the algorithm’s prediction. The predictions that were presented to the participants were accurate. Participants in the interactive condition had to enter the values of the patient’s current clinical measures before the system calculated the algorithm result. All the participants were asked to handle the information as though they were taking the described patient under their care, and the information provided was all that was available to them.

After the algorithm presented its prediction, the participants could continue to explore the UI and the information presented, and then answer whether they agreed with the algorithm’s prediction or not. After answering this question, they proceeded to the same procedure with two additional clinical cases. To avoid order bias, counterbalancing was used. The number of times participants agreed with the system’s prediction represents their reaction to the system.

### Postexperiment Questionnaires

After completing the 3 clinical cases, the participants answered 2 demographic questions about their experience and gender and 3 questionnaires about their trust in the system, the interactivity of the system, and the information relevancy of the system. The postexperiment questionnaires measured perceived understanding of the system. These consisted of the AIMQ (AIM quality) questionnaire [51] to measure information relevancy, 7 items from an interactivity questionnaire [44] that assessed interactivity, and 14 items from a questionnaire that assessed trust [30]. The latter questionnaire associated the CBT subdimensions of understandability, technical competence, and reliability from the human-computer trust questionnaire. All the questionnaires used a 7-point Likert scale (1=low and 7=high). See Table 2 for the entire list of the variables. To control for possible variance, the gender and years of experience of the participants were recorded. These analyses were performed because studies have shown a significant impact of gender [5,28,52] and years of experience [29,53] on the interaction of humans with automation, and a consequent influence on the development of human-automation trust. The questionnaire questions are presented in Multimedia Appendix 1.

**Table 2.** The experiment variables.

Construct	Scale	How it was measured
Years of experience	Continuous	Demographics
Gender	Nominal	Demographics
UI^a^ level of information relevancy	Binary	By design
UI level of interactivity	Binary	By design
Information relevancy rating	Discrete (1-7)	AIMQ^b^ questionnaire [51]
Interactivity rating	Discrete (1-7)	McMillan and Hwang [44]
Understandability	Discrete (1-7)	HCT^c^; Medsen and Gregor [30]
Technical competence	Discrete (1-7)	HCT; Medsen and Gregor [30]
Reliability	Discrete (1-7)	HCT; Medsen and Gregor [30]
Cognitive-based trust	Discrete (1-7)	HCT; Medsen and Gregor [30]
Agreement with the system	Discrete (0‐3)	Simulation software

a UI: user interface.

b AIMQ: AIM quality.

c HCT: human-computer trust.

### Data Analysis

To measure the participants’ immediate reaction to the system, the participants were grouped by the number of times they agreed with the system’s prediction. This information was compared with their information relevancy rating. Due to the different group sizes, the Welch test was used to conduct the comparisons.

A linear regression model was used to assess the influence of several variables on trust as a single construct (cognitive-based trust). Although the 2 study hypotheses aimed to identify the main effects of information relevancy and interactivity on trust, variables 1‐6 (years of experience, gender, UI level of information relevancy, UI level of interactivity, information relevancy rating, and interactivity rating) were included in the model to control for possible variance. Interactions were assessed on gender and years of experience with all the other variables.

Three linear regression models were used to assess the effect of CBT subdimensions. Variables 1‐6 were included in the models to control possible variance. Interactions were assessed on gender and years of experience with all the other variables.

## Results

### Participants’ Agreement With the System’s Prediction

**Figure 7.**
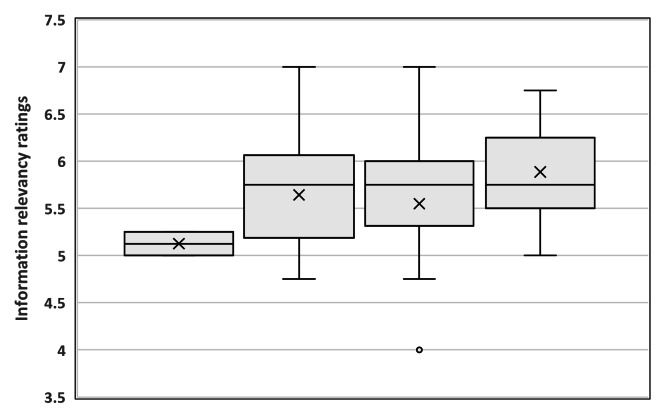
Box plot of the information relevancy ratings and the number of times participants agreed with the system’s prediction. A significant difference was found between participants who agreed with all the system’s prediction and participants who did not agree at all.

The conditions of the experiments (variables 3 and 4; Table 2) were not found to be associated with the participants’ trust in the system. However, participants’ responses to the postexperiment questionnaires reveal significant findings. Overall, the higher the participants rated information relevancy, the more frequently they agreed with the system’s prediction. Information relevancy was rated significantly higher among those who agreed 3 times with the system’s prediction compared to those who did not agree at all (*t*_11_=–3.924, 2-tailed; *P*=.05). No other comparisons between the groups were significant (see Figure 7). Participants’ agreement with the system’s prediction did not differ according to their experience, gender, or the interactivity ratings of the system.

### Trust as a Single Construct

The significant main effect for the UI level of information relevancy revealed that relevant information resulted in higher perceived trust (β=2.684; *P*=.05). Higher information relevancy ratings (β=.824; *P*<.001) and higher interactivity ratings (β=.613; *P*<.001) were associated with higher perceived trust in the system. A significant interaction between UI level of interactivity and years of experience (β=–.056; *P*=.05) revealed lower trust ratings among experienced participants with higher interactivity ratings. The adjusted *R*^2^ of the regression model was 0.5296.

### CBT Subdimensions

A significant main effect was observed for the UI level of information relevancy and technical competence (β=4.5; *P*<.001). In addition, across all the models, significant main effects for information relevancy ratings and interactivity ratings were observed. The statistical measures are summarized in Table 3. No other significant main or interaction effects were observed across the subdimensions.

**Table 3.** Statistics for the subdimensions of the cognition-based trust (CBT) dimension.

CBT subdimension	β_information relevancy ratings_	*P*_information relevancy ratings_	β_interactivity ratings_	*P*_interactivity ratings_
Technical competence	1.18	<.001	.6	<.001
Understandability	.4	<.001	.53	<.001
Reliability	.72	<.001	.53	<.001

## Discussion

### Principal Findings

Trust is difficult to measure. Participants’ agreement with the system’s prediction did not differ according to the experimental conditions. However, in the postexperiment questionnaire, higher information relevancy ratings and interactivity ratings were associated with higher perceived trust in the system, and the explicit visual presentation of the features of the ML algorithm on the user interface resulted in lower trust by the participants.

### Information Relevancy

The results of our experiment revealed that information relevancy plays an important role in operators’ trust in ML-based systems. Two different, but complementary questions were addressed and they are (1) to what extent does relevant information enhance intensivists’ trust in ML-based CDSSs? and (2) what type of information do intensivists consider to be relevant? The answer to the first question is derived directly from the results—perceived relevant information is important and affects various aspects of the operators’ trust in the system. This finding supports the first hypothesis and corroborates studies from diverse domains, which found that information relevancy substantially influences users’ trust in technological systems [41,54,55].

Regarding the second question, discerning the type of information that intensivists consider relevant is more complicated. As hypothesized, providing detailed information about the algorithm’s features decreased the participants’ trust in the system. A possible explanation for the decreased trust is that the participants found the detailed information about the ML algorithm confusing and irrelevant. Accordingly, the information about the ML algorithm may have supported the participants’ belief that they were dealing with a black box algorithm, and this, in turn, may have fostered distrust of the system [13].

Across all the CBT subdimensions assessed (understandability, technical competence, and reliability), the greater the relevancy of the information presented in the UI, according to the participants, the higher their trust. This concurs with the analysis of trust as a standalone construct and thus supports the first hypothesis.

The understandability and reliability ratings were not found to differ significantly between the information relevancy conditions. This suggests that the presentation of ML features did not significantly decrease the participants’ ratings of understandability and reliability. However, ratings of technical competence did differ between the information relevancy conditions. This could indicate a stronger effect on trust, in the technical competence subdimension, compared to understandability and reliability.

### Interactivity

The participants’ trust ratings were not found to differ significantly between conditions. However, trust ratings increased as participants’ perception of UI interactivity increased. This finding supports the second hypothesis and is in line with a meta-analysis by Yang and Shen [56], which concluded that perceived interactivity was much more effective than objective interactivity.

Two possibilities arise to explain the gap between participants’ perceptions of the interactivity and the actual UI level of interactivity. First, within the 2 interactivity levels, the objective gap between the different conditions may not have been strong enough. The less interactive condition also forced 2-way communication between the participants and the UI. Possibly, the initial user engagement did not add enough interactivity to render a noticeable difference. Alternatively, the participants may not have perceived increased interactivity. Second, although entering and copying values to and from the patient record is a common task clinicians must apply in a subset of the IT systems in the ICU, participants may have considered that manually entering the patient’s clinical measures was dull or redundant. This could have reduced participants’ opinion of the system and led to lower trust ratings.

Although more interactive perceptions of the UI were associated with higher trust ratings, it is arguable whether extreme levels of interactivity are always preferable. Kalet et al [57] investigated the influence of different interactivity levels in a computer-assisted instruction system on medical students’ performances. They found that a mid-range UI level of interactivity maximized improvements in the performance of clinical skills. Yang and Shen [56] found that extremely high levels of website interactivity were less effective than moderate levels. However, pinpointing the exact amount of moderate interactivity, universally or specifically for a domain, is challenging. Furthermore, treating interactivity as a continuous variable and fitting it into a linear regression model could lead to measurement and interpretation errors. According to Yang and Shen [56], interactivity should be considered as a curvilinear variable, with the peak at the center of the curve and not at the edges. When fitting a linear regression model to an interactivity variable, the latter is considered linear, but this is not always the case. This approach may fail to capture the real influence of different levels of interactivity.

Across the 3 CBT dimensions examined (understandability, technical competence, and reliability), the more interactive the UI, according to the participants’ perception, the higher their trust. This was precisely the situation when trust was analyzed as a standalone construct. Otherwise, the interactivity levels examined were not found to differ between the CBT dimensions. Notably, a linear regression model was set for each subdimension. Although the results showed that the more interactive the UI, the higher the ratings for each subdimension, moderate levels of interactivity may have had a greater effect on those subdimensions.

Finally, the literature is scant regarding correlations between experience and interactivity, and additional research is needed to elaborate on the significant negative interaction across years of experience and interactivity ratings.

### Limitations and Future Research

Some limitations of this study represent opportunities for future research. First, the study design, limited resources, and the period the study was conducted (between the first and second waves of the COVID-19 pandemic) posed limitations on participant recruitment. The limited sample size dictated a design with only 2 levels of each variable. Future research should explore advanced and more realistic UI interactions and different information types. Second, although Madsen and Gregor’s [30] approach was used to analyze trust, the ABT dimensions were not explored. Such investigation is needed to obtain a wider view of the relations between trust and its subdimensions, both cognitive-based and affect-based. Third, due to time limitations, the study did not evaluate participants’ attitudes and changes in trust in the system over time. Finally, the study was performed in a simulation environment, using a specific interface design, and using case studies rather than real-time data from patients. Investigating clinician collaboration with a variety of interface designs, within real-world information systems used in diverse health care settings could yield a deeper understanding of future interface design.

### Conclusions

Developing ML algorithms is only the first step toward improving medical treatment. To increase acceptance and trust of ML-based CDSSs, and expand their use, a broader and more multidisciplinary approach (eg, user-centered design) should be taken. This approach needs to be specifically evaluated in the health care work environment, considering its unique challenges and professional personnel. A better understanding of means to increase intensivists’ trust in ML-based CDSSs may open new opportunities for user-centered design and improved decision-making processes in the ICU.

Human factor studies, like this one, highlight the importance of understanding the effect of specific UI features when designing ML-based CDSS and other “artificial intelligence” systems. This study focused on the effects of 2 UI features related to intensivists’ trust in ML-based CDSSs. We demonstrated that the level of relevancy of the information that is presented in the UI and the interactivity level of the UI can play major roles when designing ML-based CDSSs. However, to enhance trust in these systems, more UI features should be investigated.

A wide point of view on trust should be maintained. In this study, trust as a standalone construct was influenced significantly by the different information relevancy levels in the tested conditions. Of the CBT subdimensions, only technical competence was influenced in the same way. These findings emphasize the need to analyze trust from different perspectives. For the research community and system designers, this may promote a broad understanding of means to enhance and foster trust in ML-based CDSSs, as well as in other “artificial intelligence” systems.

## Supplementary material

10.2196/56924 Multimedia Appendix 1 The questionnaire.

## Abbreviations

ABT: affect-based trust
AIMQ: AIM quality
CBT: cognition-based trust
CDSS: clinical decision support system
HCI: human-computer interaction
ICU: intensive care unit
ML: machine learning
UI: user interface
