# Supplementary material for: The Impact of Information Relevancy and Interactivity on Intensivists’ Trust in a Machine Learning–Based Bacteremia Prediction System: Simulation Study
Source: JMIR Hum Factors. 2024 Aug 1;11:e56924. doi: 10.2196/56924 (PMC11310737; doi:10.2196/56924)
Supplement: Multimedia Appendix 1 [file humanfactors-v11-e56924-s001.docx]

**The questionnaire (the order of the questions was mixed)**

All the questionnaires utilized a seven-point Likert scale (agree…Somewhat agree…Neither agree nor disagree…Somewhat disagree…Disagree…Strongly disagree)

Q1: The Bacteremia prediction software always provides the advice I require to make my decision

Q2: The Bacteremia prediction software performs reliably

Q3: The Bacteremia prediction software responds the same way under the same conditions at different times

Q4: I can rely on the Bacteremia prediction software to function properly

Q5: The Bacteremia prediction software analyzes problems consistently

Q6: The Bacteremia prediction software uses appropriate methods to reach decisions

Q7: The Bacteremia prediction software has sound knowledge about this type of problem built into it

Q8: The advice the Bacteremia prediction software produces is as good as that which a highly competent person could produce

Q9: The Bacteremia prediction software makes use of all the knowledge and information available to it to produce its solution to the problem

Q10: I know what will happen the next time I use the Bacteremia prediction software because I understand how it behaves

Q11: I understand how the Bacteremia prediction software will assist me with decisions I have to make

Q12: Although I may not know exactly how the Bacteremia prediction software works, I know how to use it to make decisions about the problem

Q13: It is easy to follow what the Bacteremia prediction software does

Q14: I recognize what I should do to get the advice I need from the Bacteremia prediction software the next time I use it

Q15: The Bacteremia prediction software enables two-way communication

Q16: The Bacteremia prediction software is interactive

Q17: The Bacteremia prediction software enables conversation

Q18: The Bacteremia prediction software facilitates variety of content

Q19: The Bacteremia prediction software keeps my attention

Q20: It was easy to find my way through the Bacteremia prediction software

Q21: The Bacteremia prediction software lacks content

Q22: This information presented in the Bacteremia prediction software is useful to my work

Q23: The information presented in the Bacteremia prediction software is relevant to my work

Q24: The information presented in the Bacteremia prediction software is appropriate for my work

Q25: The information presented in the Bacteremia prediction software is applicable for my work
